# Real-time effects of PEEP and tidal volume on regional ventilation and perfusion in experimental lung injury

**DOI:** 10.1186/s40635-020-0298-2

**Published:** 2020-02-21

**Authors:** João Batista Borges, John N. Cronin, Douglas C. Crockett, Göran Hedenstierna, Anders Larsson, Federico Formenti

**Affiliations:** 1 Centre for Human and Applied Physiological Sciences, King’s College London, London, UK; 2 Nuffield Division of Anaesthetics, University of Oxford, Oxford, UK; 3 Hedenstierna Laboratory, Department of Medical Sciences, Uppsala University, Uppsala, Sweden; 4 Hedenstierna Laboratory, Department of Surgical Sciences, Uppsala University, Uppsala, Sweden

**Keywords:** Respiratory distress syndrome, Adult, Mechanical ventilation, Electrical impedance tomography, Pulmonary circulation, Ventilation-perfusion ratio

## Abstract

**Background:**

Real-time bedside information on regional ventilation and perfusion during mechanical ventilation (MV) may help to elucidate the physiological and pathophysiological effects of MV settings in healthy and injured lungs. We aimed to study the effects of positive end-expiratory pressure (PEEP) and tidal volume (*V*_T_) on the distributions of regional ventilation and perfusion by electrical impedance tomography (EIT) in healthy and injured lungs.

**Methods:**

One-hit acute lung injury model was established in 6 piglets by repeated lung lavages (*injured* group). Four ventilated piglets served as the *control* group. A randomized sequence of any possible combination of three *V*_T_ (7, 10, and 15 ml/kg) and four levels of PEEP (5, 8, 10, and 12 cmH_2_O) was performed in all animals. Ventilation and perfusion distributions were computed by EIT within three regions-of-interest (ROIs): nondependent, middle, dependent. A mixed design with one between-subjects factor (group: *intervention* or *control*), and two within-subjects factors (PEEP and *V*_T_) was used, with a three-way mixed analysis of variance (ANOVA).

**Results:**

Two-way interactions between PEEP and group, and *V*_T_ and group, were observed for the dependent ROI (*p* = 0.035 and 0.012, respectively), indicating that the increase in the dependent ROI ventilation was greater at higher PEEP and V_T_ in the injured group than in the control group. A two-way interaction between PEEP and *V*_T_ was observed for perfusion distribution in each ROI: nondependent (*p* = 0.030), middle (*p* = 0.006), and dependent (*p* = 0.001); no interaction was observed between injured and control groups.

**Conclusions:**

Large PEEP and *V*_T_ levels were associated with greater pulmonary ventilation of the dependent lung region in experimental lung injury, whereas they affected pulmonary perfusion of all lung regions both in the control and in the experimental lung injury groups.

## Introduction

Real-time information on regional ventilation and perfusion, and their changes, during mechanical ventilation (MV) may help elucidate the physiological and pathophysiological effects of MV settings in healthy and injured lungs.

A single-compartment model of the healthy lung can describe aeration and its changes associated with a homogeneous distribution of airway pressures, expansion, and stretching across the lung parenchyma [1]. The positive airway pressure applied during MV may affect pulmonary ventilation and perfusion distributions, and their tidal changes, especially in the presence of acute lung injury.

The effects of positive end-expiratory pressure (PEEP) on the regional distribution of tidal volume (*V*_T_) and recruitment have been investigated by chest computed tomography in sedated-paralyzed patients with the acute respiratory distress syndrome (ARDS) [2]. It was evidenced that PEEP made the gas distribution more homogeneous, stretching the upper levels and recruiting the lower ones [2].

Electrical impedance tomography (EIT) has emerged as a new functional-imaging method potentially meeting many clinical needs. It is a non-invasive, radiation-free tool to monitor, in real-time and at the bedside, the distribution of pulmonary ventilation [3–11]. Subjecting the patient’s chest to minute electrical currents, EIT measures the electric potentials at the chest wall surface to produce two-dimensional (2D) images that reflect the impedance distribution within the thorax. Cyclic variations in pulmonary air and blood content are the major determinants for the changes in thoracic impedance. Because cyclic changes in local impedance mainly correspond to changes in lung aeration, EIT can reliably assess imbalances in the distribution of regional ventilation [3, 4, 11, 12]. Besides other features like portability and the possibility of around-the-clock monitoring, the high temporal resolution (modern EIT devices generate up to 50 images per second) is another important aspect of this imaging method, which also allows the study of rapid physiological phenomena, such as the regional perfusion [13].

We aimed to measure pulmonary regional ventilation and perfusion at 12 combinations of PEEP and *V*_T_ levels in piglets with saline lavage-induced lung injury and in mechanically ventilated control piglets. We sought to determine (1) if the PEEP and *V*_T_ effects on the regional distribution of tidal volume in injured lungs are also detectable by EIT; (2) whether there are also effects on perfusion; and (3) does the presence or absence of injury affect PEEP- or *V*_T_-related changes in ventilation and perfusion distributions.

## Materials and methods

The study was performed at the Hedenstierna Laboratory, Uppsala University. The Regional Animal Ethics Committee approved the study.

Eleven piglets (2–3 months old, weight 30.7 ± 1.5 kg, mean ± SD) of mixed Hampshire, Yorkshire, and Swedish country breeds were included in the study. All animals were studied lying in the supine position under general anesthesia with mandatory-mode mechanical ventilation provided via tracheostomy. They were pre-medicated with intramuscular xylazine 2 mg/kg, ketamine 20 mg/kg, and midazolam 0.5 mg/kg. An ear vein was cannulated and intravenous ketamine 32 mg/kg/h, fentanyl 4 mcg/kg/h, and midazolam 0.16 mg/kg/h were administered. Adequacy of anesthesia was confirmed by the absence of reaction to painful stimulation between the front hooves and the absence of any signs of sympathetic stimulation after paralysis. Muscle relaxation was achieved using continuous infusion of rocuronium titrated against the spontaneous respiratory effort. Normovolemia was maintained with intravenous infusion of Ringer’s lactate solution at 20 ml/kg/h for the first hour followed by 10 ml/kg/h.

MV was performed using a Servo-I ventilator (Maquet, Rastatt, Germany). During the instrumentation phase, ventilation was provided in volume-controlled mode, with *V*_T_ of 10 ml/kg, respiratory rate (RR) 25 breaths/min, PEEP 5 cm H_2_O, inspiratory-to-expiratory (I:E) ratio 1:2, and fraction of inspired oxygen (F_I_O_2_) 0.4. ECG, invasive systemic, central venous, and pulmonary artery blood pressures were transduced using a standard clinical monitor (IntelliVue M8004A, Philips Healthcare, Best, Netherlands). Digital outputs from the ventilator and clinical monitor were continuously recorded using the acqIS software (EPiQ Life Science AB, Kista, Sweden). A femoral artery was cannulated for pulse contour cardiac output monitoring (PiCCO, Pulsion Medical Systems, Munich, Germany). Pulmonary artery flotation catheter thermodilution cardiac output measurements and arterial blood gas analyses were performed at the beginning and end of the scanning series for each animal.

### Acute lung injury model

Following baseline measurements, a one-hit acute lung injury model was established in 7 randomly chosen animals (injured group), with repeated lung lavages (30 ml/kg) of isotonic saline applied until an arterial partial pressure of O_2_ and fraction of inspired oxygen ratio (PaO_2_/F_I_O_2_) of 200 mmHg was reached. During the lavages, mechanical ventilation was set in pressure-controlled mode, with F_I_O_2_ 1.0, RR 30, PEEP 5, and a driving pressure resulting in a *V*_T_ of 6 ml/kg. If required, an infusion of noradrenaline (0.01 to 0.1 mcg/kg/min) was commenced following an injury to maintain adequate mean arterial blood pressure.

### Investigational protocol

We studied the effects of combinations of PEEP and *V*_T_ on regional ventilation and perfusion by EIT in volume-controlled mode. A randomized sequence of any possible combination of three *V*_T_ (7, 10, and 15 ml/kg) and four levels of PEEP (5, 8, 10, and 12 cmH_2_O) was performed. In total, 12 conditions were studied.

### Electrical impedance tomography

Pulmonary EIT data were recorded at 50 Hz with 32 electrodes equidistantly placed around the circumference of the thorax just below the level of the axilla (Enlight, TIMPEL SA, São Paulo, Brazil) [13, 14]. The following functional images were generated by EIT:

1. Ventilation maps derived from relative impedance changes, which reliably track local, pixel-by-pixel changes in the content of air within the lung [12, 15]. It is expressed as the percentage of total pulmonary ventilation through each of the three ROIs (total 100%).
2. Perfusion maps obtained by injecting a bolus of 10 ml of a hypertonic solution (NaCl 10%) into a central venous catheter during an expiratory breath hold for 20 s. Due to its high conductivity, NaCl 10% acts as an EIT contrast agent [16], which after injection into the right atrium during apnea passes through the pulmonary circulation, thereby producing a dilution curve that follows typical first-pass kinetics. The resulting regional impedance curves are then analyzed to quantitatively assess regional perfusion [13, 17, 18], expressed as the percentage of total pulmonary blood flow through each of the three ROIs (total 100%).

**Fig. 1:**
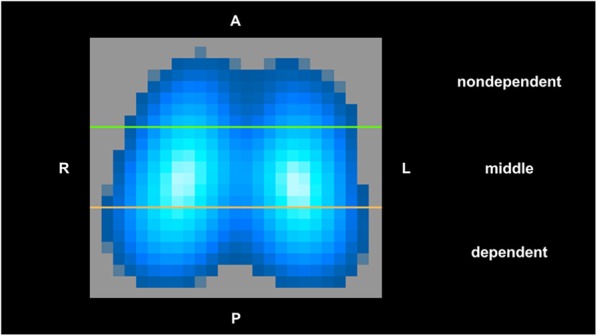
Representative image of regional distribution of pulmonary ventilation as recorded by electrical impedance tomography (EIT) in one piglet from the control group. Three regions-of-interest (ROIs) of the same vertical height were constructed from top (anterior) to bottom (posterior) of the lung: nondependent, middle, and dependent ROI. In this ventilation map, lighter blue indicates greater ventilation than darker blue, with white representing the greatest ventilation. A = anterior; P = posterior; L = left; R = right

For the quantitative analysis of the ventilation and perfusion distributions by EIT, the lungs were sub-segmented into three isogravitational regions-of-interest (ROIs): nondependent, middle, and dependent regions (Fig. 1).

### Control group

The same EIT imaging protocol and data analysis were performed in four animals, which did not receive saline lavages.

### Statistics

The Shapiro-Wilk test was used to test data for normality. A mixed design with one between-subjects factor (group: intervention or control), and two within-subjects factors (PEEP and *V*_T_) was used, with a three-way mixed analysis of variance (ANOVA). The Bonferroni adjustment for multiple tests was applied for post hoc comparisons. The statistical analyses were conducted with SPSS (version 20; IBM Corp, IBM SPSS Statistics for Windows, Armonk, NY). Individual *p* values to indicate statistical tests’ significance are reported were relevant. Values presented are mean and SEM unless otherwise stated.

## Results

The fatality rate was 1/11 piglets, due to a cardiovascular event (this was in the injured group, thus 4 controls and 6 injured completed the study). The 10 piglets that survived the whole experiment were included in the analysis.

Just before starting the application of the twelve combinations of PEEP and *V*_T_, cardiopulmonary parameters were measured (Table 1). During the measurements of these cardiopulmonary parameters, mechanical ventilation was set in volume-controlled mode, with F_I_O_2_ 0.3–0.4 for the control animals and 0.7–0.8 for those with lung injury, RR 25, PEEP 5, and a *V*_T_ of 10 ml/kg.

**Table 1:** Cardiopulmonary parameters

Parameter	Control				Injured						Control	Injured
	C1	C2	C3	C4	I1	I2	I3	I4	I5	I6	Mean (SD)	Mean (SD)
Weight (kg)	30	33	30	33	30	28	29	33	31	31	31 (2)	30 (2)
Lavage volume (L)	0	0	0	0	4	5	2	4	9	2	0 (0)	4 (3)
P/F ratio (mmHg)	287	384	441	373	158	206	153	104	164	143	371 (64)	154 (33)*
ABP (mmHg)	65	78	89	79	68	95	97	97	75	62	78 (10)	82 (16)
PAP (mmHg)	20	27	14	20	34	27	27	20	32	34	20 (5)	29 (6) #
CVP (mmHg)	6	11	11	6	13	8	11	6	8	12	9 (3)	10 (3)
HR (bpm)	75	87	82	143	97	105	98	107	107	93	97 (31)	101 (6)
CO (L/min)	1.9	4.0	3.0	6.1	3.6	3.9	3.2	4.0	4.1	3.4	3.8 (1.8)	3.7 (0.4)

*Abbreviations*: *C* Control, *I* Injured (C1 to C4 and I1 to I6 refer to different animals), *P/F ratio* PaO_2_/F_I_O_2_ ratio sampled at PEEP 5 cmH_2_O, *ABP* mean arterial blood pressure, *PAP* Pulmonary arterial pressure, *CVP* Central venous pressure, *HR* Heart rate, *CO* Cardiac output measured by pulmonary artery flotation catheter thermodilution

*p < 0.001

^#^*p* = 0.02 from independent samples *t* test

Data are presented as mean (SD), where appropriate

**Fig. 2:**
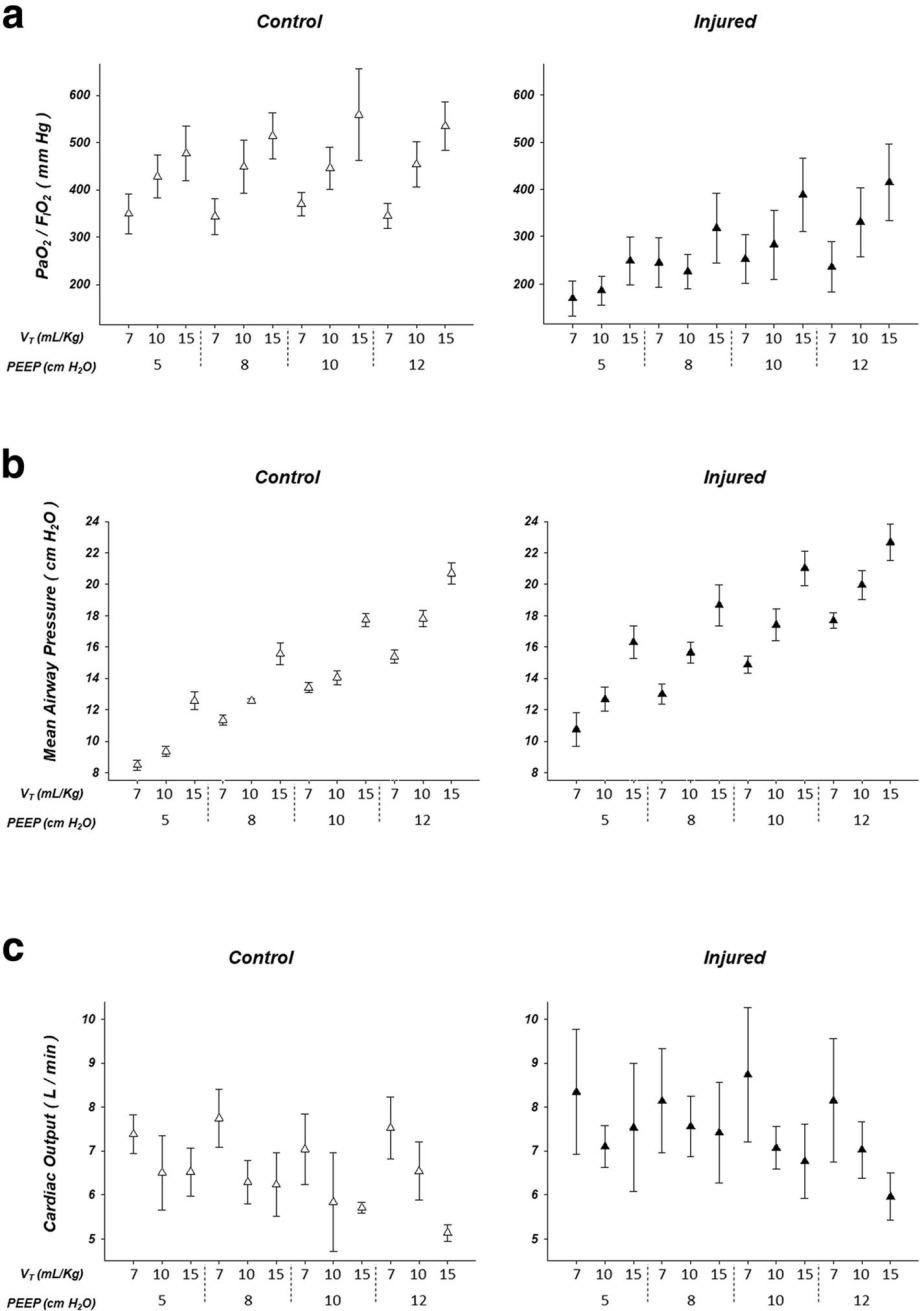
Arterial partial pressure of O_2_ and fraction of inspired oxygen ratio (PaO_2_/F_I_O_2_; **a**), mean airway pressure (**b**), and cardiac output (**c**) in the control (left, n = 4) and injured (right, n = 6) groups. PEEP = positive end-expiratory pressure. *V*_T_ = tidal volume

Figure 2 shows arterial partial pressure of O_2_ and fraction of inspired oxygen ratio (2A), mean airway pressure (2B), and cardiac output (2C) in the control (left) and injured (right) groups, in the 12 combinations of PEEP and *V*_T_.

**Fig. 3:**
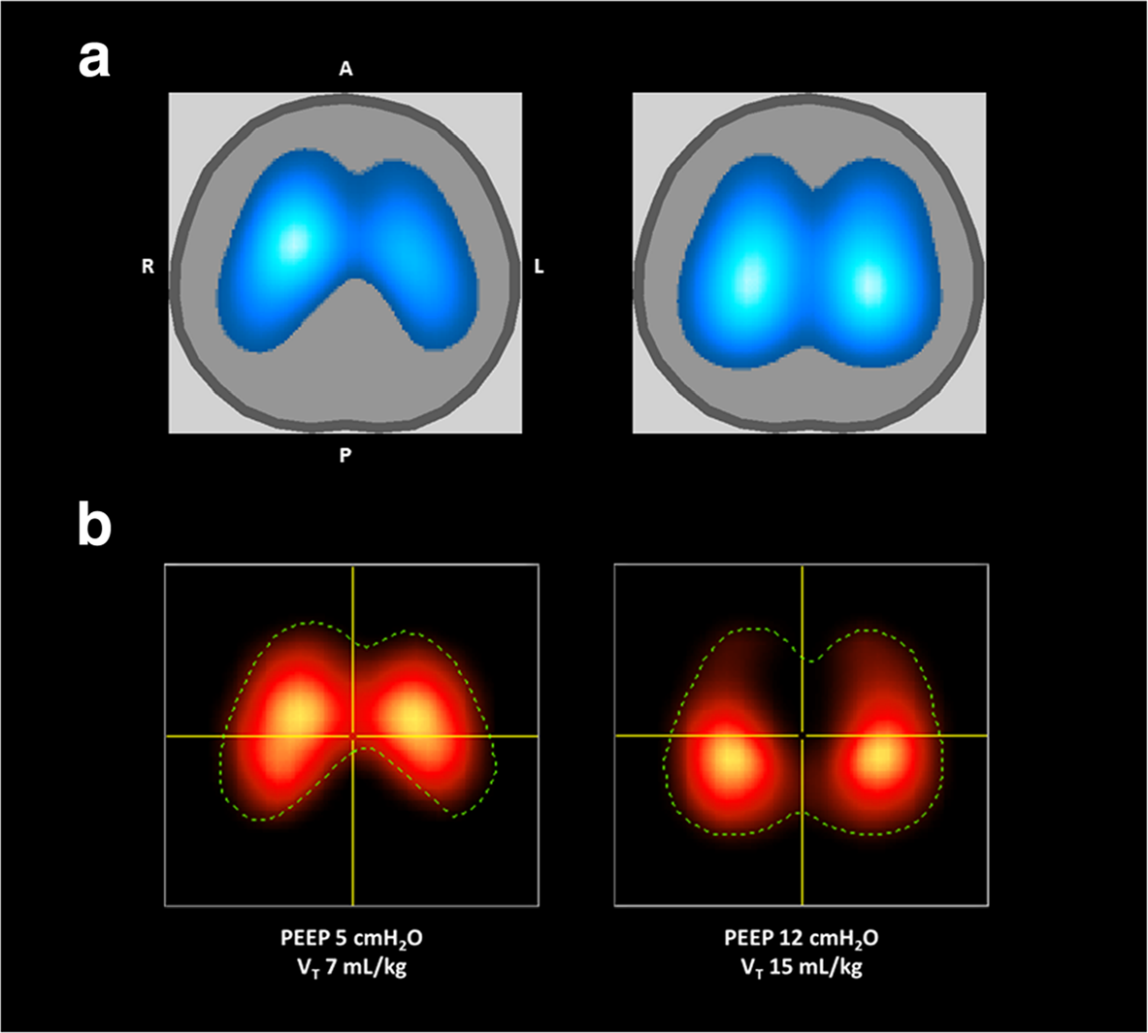
Representative images of regional distribution of pulmonary ventilation (**a**) and perfusion (**b**) at two different PEEP and *V*_T_ levels as recorded by electrical impedance tomography (EIT) in one piglet from the injured group. In the ventilation maps (**a**), lighter blue indicates greater ventilation than darker blue, with white representing the greatest ventilation. Similarly, in the perfusion maps (**b**), the lighter red indicates greater perfusion than darker red, with yellow indicating the greatest perfusion. The dotted line in the perfusion maps shows the contour of the corresponding ventilation map (i. e., the corresponding pulmonary ventilation area studied at the same point in time, hence under the same mechanical ventilation settings). PEEP = positive end-expiratory pressure. *V*_T_ = tidal volume. A = anterior; P = posterior; L = left; R = right (all images)

Figure 3 shows representative EIT functional images illustrating the differences in the distribution of pulmonary ventilation and perfusion at two levels of PEEP and *V*_T_.

### Regional ventilation

**Fig. 4:**
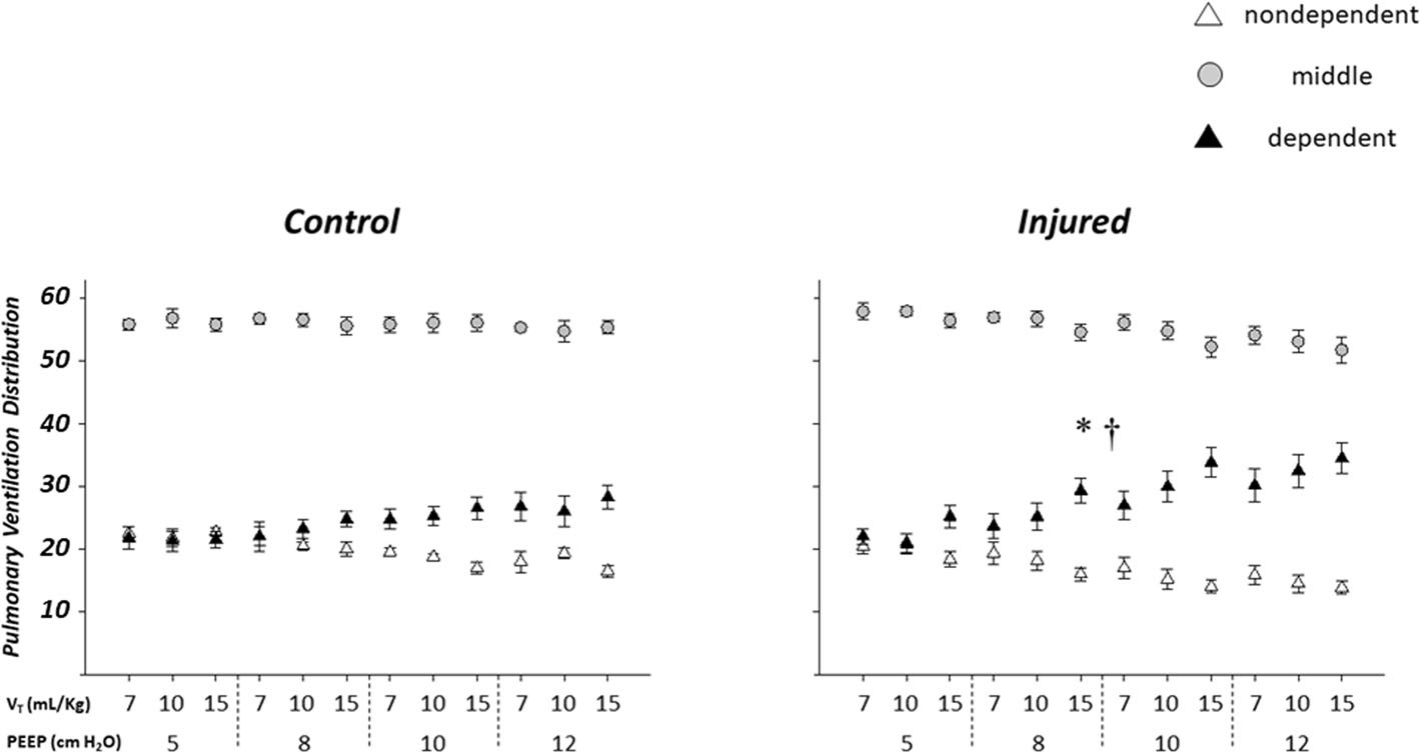
Regional distribution of pulmonary ventilation in the control (left, n = 4) and injured (right, n = 6) groups. Larger PEEP levels were associated with greater percent increase in the ventilation of the dependent lung in the injured group than in the control group. Similarly, larger *V*_T_ was associated with a greater percent increase in dependent lung ventilation in the injured group than in the control group. * indicates greater percent increase in the ventilation of the dependent lung region with larger PEEP in the injured than in the control group; † indicates greater percent increase in the ventilation of the dependent lung region with larger *V*_T_ in the injured than in the control group. PEEP = positive end-expiratory pressure. *V*_T_ = tidal volume

Figure 4 shows the distribution of regional pulmonary ventilation at different PEEP and *V*_T_ levels. Higher PEEP and *V*_T_ levels were associated with greater percent ventilation in the dependent ROI in the injured group than in the control group.

A two-way interaction between PEEP and group (injured/control) was observed for the dependent ROI (*p* = 0.035), with PEEP-related increase in dependent ROI ventilation being greater in the injured than in the control group.

A two-way interaction between *V*_T_ and group (injured/control) was also observed for the dependent ROI (*p* = 0.012), indicating that the increase in the dependent ROI ventilation was greater at higher *V*_T_ in the injured group than in the control group.

For instance, at PEEP 12 cmH_2_O with *V*_T_ 15 ml/kg the dependent ROI ventilation in the injured group was 34.5 ± 5.6% versus 22.0 ± 2.4% at PEEP 5 cmH_2_O with V_T_ 7 ml/kg.

### Regional perfusion

No interactions between PEEP and group (injured/control) and/or between *V*_T_ and group (injured/control) were observed for regional perfusion.

**Fig. 5:**
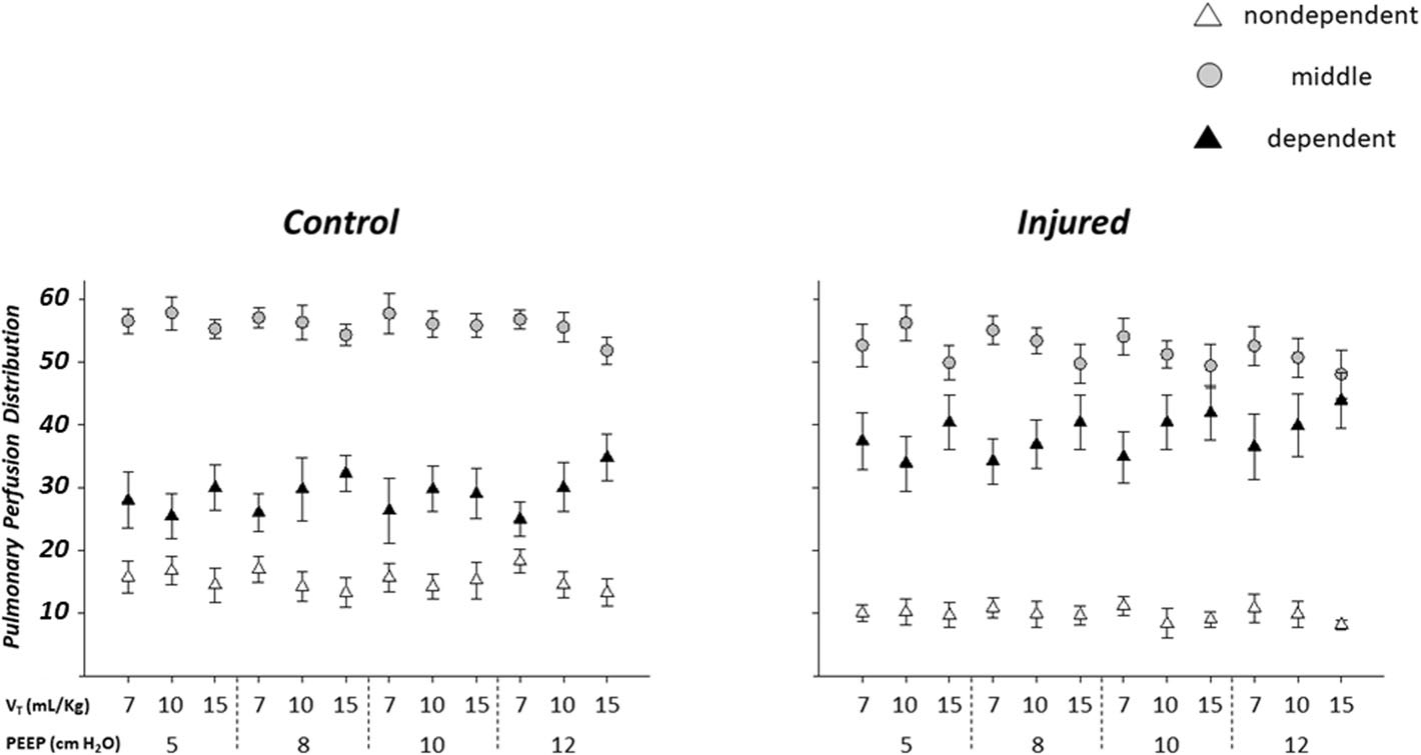
Regional distribution of pulmonary perfusion in the control (left, n = 4) and injured (right, n = 6) groups. Both larger PEEP and larger V_T_ determined a significant perfusion change in each of the ROIs considered: nondependent (*p* = 0.030), middle (*p* = 0.006), and dependent (*p* = 0.001). PEEP = positive end-expiratory pressure. *V*_T_ = tidal volume

Figure 5 shows the distribution of regional pulmonary perfusion at different PEEP and *V*_T_ levels. A two-way interaction between PEEP and *V*_T_ was observed for each ROI: nondependent (*p* = 0.030), middle (*p* = 0.006), and dependent (*p* = 0.001).

For instance, pooling together the two groups, at PEEP 5 cmH_2_O with *V*_T_ 7 ml/kg versus PEEP 12 cmH_2_O with *V*_T_ 15 ml/kg the nondependent ROI perfusion was 12.6 ± 4.6% versus 10.2 ± 3.7%, the middle ROI perfusion was 54.3 ± 5.9% versus 49.5 ± 7.2%, and the dependent ROI perfusion was 33.2 ± 9.6 % versus 40.2 ± 9.7%.

## Discussion

Regional ventilation of the most dependent lung region was greater with larger PEEP and tidal volume in piglets with saline lavage-induced lung injury. In contrast, pulmonary blood flow of all lung regions was altered by larger PEEP and tidal volume both in control piglets and in piglets with saline lavage-induced lung injury.

Our study demonstrates that EIT can measure physiological and supra-physiological levels of pulmonary ventilation and perfusion, and their changes associated with different MV settings such as PEEP and *V*_T_, continuously, in real-time, and at the bedside. This information may be critical for a timely evaluation of the precision and efficacy of attempts to minimize some of the main ventilator-induced lung injury (VILI) mechanisms, particularly in the presence of alveolar collapse, common in the lungs of patients with the ARDS [19].

Our data highlight that airspaces collapse in the injured group altered the pulmonary regional ventilation response to different PEEP and *V*_T_ levels. Notwithstanding, when analyzing the effects of PEEP and *V*_T_ on regional perfusion, airspaces collapse did not seem to play a major role. Regarding regional perfusion, the mutual interaction between PEEP and tidal volume seemed to be more relevant. Our findings on regional ventilation may be interpreted as predominantly gravity-related, since the ventilation of the more dependent region exhibited a greater increase in its percentage with increasing *V*_T_ and PEEP in the injured group than in the control one. On the other hand, our findings on regional perfusion, where all regions were altered and the presence or not of lung injury did not have an effect, suggest that *V*_T_ and PEEP interacted all over the lung parenchyma on the redistribution of regional perfusion to the dependent zones [20, 21], also in agreement with pulmonary blood volume tidal redistribution [22].

Pulmonary perfusion can be affected by anesthesia and by mechanical ventilation, affecting arterial blood oxygenation [23–27]. The greatest determinant of pulmonary blood perfusion distribution is the difference between alveolar and pulmonary capillary pressures [28]. Perfusion increases down the gravitational gradient in the lung, due to hydrostatic forces and structural factors. Atelectasis is pronounced in the dependent lung regions, where perfusion of non-ventilated lung parenchyma produces a shunt effect of around 8–10% of cardiac output in healthy lungs [24], and a greater shunt in injured lungs. In addition, non-gravitational inhomogeneity of perfusion can reduce arterial blood oxygenation. Elevated airway pressures applied to the lung during mechanical ventilation can compromise venous return and redirect blood flow to dependent lung regions, with the effect being more marked at higher levels of PEEP and/or *V*_T_ [29]. Our data demonstrate that these effects can be regionally monitored, measured, and tracked in real-time and at the bedside by EIT.

Our distributions of regional pulmonary perfusion at different PEEP and *V*_T_ levels may have been also partly influenced through hypoxic pulmonary vasoconstriction (HPV) [30]. In conditions of atelectasis, as in the injured group here, HPV could have been greater at low levels of PEEP and *V*_T_. Noteworthy, we have shown before that perfusion is similar, whether a region is collapsed is aerated but non-ventilated [13], findings that fit with observations by Benumof [31]—that HPV is the main mechanism of reduced blood flow in atelectatic regions, not mechanical obstruction.

The main limitation of our study may be in the animal model that is not capable of reproducing all of the key characteristics of lung injury in humans, for example in ARDS patients, where EIT titration of MV may be most helpful. Any animal model is relevant for only limited aspects of ARDS pathophysiology. However, if the specific characteristics of an animal model are carefully taken in account, and its findings are interpreted in the context of the study limitations, animal investigations can provide valid assessments of relevant elements of ARDS in human patients. The surfactant depletion by saline lavage model was developed based on the observation that ARDS is associated with depletion of surfactant from the air spaces and reduced concentrations of surfactant-associated proteins in bronchoalveolar lavage fluid [32]. However, determining precisely the extent to which the lung injury is caused by the saline lavage, by mechanical ventilation, or both remains challenging. Depletion of surfactant may be associated with lung injury via two mechanisms: greater lung collapse and increased likelihood of mechanical injury during repeated cycles of airspaces opening/closure, and impaired alveolar host defenses. Characteristically, saline lavage leads to almost immediate hypoxemia, which may be rapidly reversed by recruitment maneuvers, suggesting that the gas exchange abnormalities reflect collapsed alveoli with otherwise intact alveolar walls. The saline lavage by itself has little consequence in terms of permeability changes or inflammation [33], although TNF-alpha is detectable in lavage fluid. Despite surfactant depletion being an important feature of ARDS in humans, it usually appears as a consequence rather than the primary cause of lung injury [34]. In ARDS, surfactant abnormalities occur because of injury to the alveolar epithelium and exudation of protein-rich edema fluid into the alveolar spaces. Saline lavage of the lungs results in surfactant depletion in the absence of major alveolar epithelial damage. Epithelial damage occurs only when the saline lavage is followed by an injurious ventilatory strategy. Therefore, surfactant depletion followed by mechanical ventilation simulates established ARDS and provides information about the consequences of surfactant depletion, but it is less useful in modeling the initial pathophysiological mechanisms that lead to ARDS. The major advantage of the saline lavage model is that it provides an ideal way to test the effects of different ventilatory strategies on the development of lung injury [33].

The approach applied in this study for the determination of regional pulmonary perfusion by EIT presents some limitations. The regional time-impedance curves resulting from 10% NaCl injection [16, 35, 36] were fitted on a pixel-by-pixel basis by a gamma-variate function to quantitatively assess regional perfusion [17, 18]. The subtraction of the cardiac component of perfusion from mixed pixels while maintaining the net lung component by using an EIT gamma variate algorithm is an essential feature of this method. Such was made possible by the fitting also of the early cardiac component, also on a pixel-by-pixel basis by a corresponding gamma-variate function. However, at the edges of the heart, some challenging overlap between the behavior of lung and heart tissue may have remained, sometimes causing uncertainties in the double-function fitting process applied. Although the location of the cardiac chambers in humans could make this differentiation easier in the clinical setting, more studies on this subject are needed.

The maximal slope method applied is based on the assumption that no tracer leaves the ROI before the peak artery concentration is reached. Such assumption could be violated in the presence of the combination of high blood flow and low blood volume, leading to small estimation errors, provided that blood volume decreases by no more than one order of magnitude.

It is possible that some sodium chloride diffused outside the pulmonary blood vessels. In this situation, the solute that remains in the vessel will leave the lungs through the venous drainage while the diffused solute will tend to stay in the lungs, violating the conservation of mass principle (unless an extravascular compartment is accounted for). For the calculations of the maximal slope; however, the conservation of mass could still be applied correctly, since one of the assumptions is that there is no outflow of hypertonic saline before the peak of the pulmonary artery input function. In this case, the conservation of mass implies that all the solute that reaches the ROI (feeding vessel and extravascular compartment together), irrespective of whether it remains inside the vessels or not, came through the feeding artery.

EIT imaging has 2D and 3D features (the electrodes are placed within a single plane, but the finite mesh is 3D). Although reasonably large, the thickness of the EIT cross-sectional slice (~ 15 cm) may represent different proportions of the lungs, depending on the size and shape of the animal [4], without guarantee that most of the lung is represented in all animals, unless other imaging techniques are employed in parallel [37]. Finally, due to the low spatial resolution of EIT, the maximum slope time point might be slightly displaced (in time) among sub-regions within the ROI, likely causing inaccurate estimates of spatially averaged maximum slopes. The extent of this potential limitation deserves future studies.

## Conclusions

Our findings suggest that ventilation of the most dependent lung region is greater with larger levels of PEEP and tidal volume in piglets with saline lavage-induced lung injury. Moreover, pulmonary blood flow of all lung regions is affected by larger levels of PEEP and tidal volume, both in control animals and in piglets with saline lavage-induced lung injury. They also suggest that EIT is a promising bedside and noninvasive clinical tool for continuous and real-time monitoring of pulmonary ventilation that can be especially useful in severe mechanically ventilated patients such as those with ARDS. EIT can help to optimize mechanical ventilation settings, detect complications such as derecruitment, and provide estimates of perfusion distribution. More clinical validation studies are awaited to explore the full potential of the technology.

## Data Availability

The datasets used and/or analyzed during the current study are available from the corresponding author on reasonable request.

## Abbreviations

ANOVA: Analysis of variance
ARDS: Acute respiratory distress syndrome
EIT: Electrical impedance tomography
F_I_O_2_: Fraction of inspired oxygen
HPV: Hypoxic pulmonary vasoconstriction
I:E: Inspiratory-to-expiratory ratio
MV: Mechanical ventilation
PaO_2_/F_I_O_2_: Arterial partial pressure of O_2_ and fraction of inspired oxygen ratio
PEEP: Positive end-expiratory pressure
ROIs: Regions-of-interest
RR: Respiratory rate
VILI: Ventilator-induced lung injury
*V*_T_: Tidal volume
